# 4-Hydr­oxy-2,2,6,6-tetra­methyl­piperidinium trichloro­acetate

**DOI:** 10.1107/S1600536808005369

**Published:** 2008-02-29

**Authors:** Peng-Wei Zhang, Tong-Yun Zhang, Li Zhang, Yi Deng

**Affiliations:** a School of Pharmaceutical Science & Technology, Tianjin University, Tianjin 300072, People’s Republic of China; b Department of Pharmaceutics, Medical College of Chinese People’s Armed Police Force, Tianjin 300162, People’s Republic of China; c Darentang Pharmaceutical Factory, Tianjin Zhongxin Pharmaceutical Group Co. Ltd, Tianjin 300457, People’s Republic of China

## Abstract

In the crystal structure of the title compound, C_9_H_20_NO^+^·Cl_3_CCOO^−^, the cations and anions are connected via O—H⋯O, N—H⋯O, O—H⋯Cl and N—H⋯Cl hydrogen bonding. The six-membered ring adopts a chair conformation with the hydroxyl group in an equatorial position.

## Related literature

For related literature, see: Borzatta & Carrozza (1991 ▶).
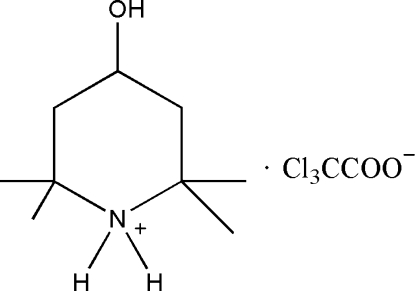


## Experimental

### 

#### Crystal data

C_9_H_20_NO^+^·C_2_Cl_3_O_2_^−^
*M* *_r_* = 320.63
Monoclinic, 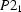

*a* = 6.3468 (13) Å
*b* = 14.450 (3) Å
*c* = 8.2175 (16) Å
β = 95.19 (3)°
*V* = 750.5 (3) Å^3^
*Z* = 2
Mo *K*α radiation
μ = 0.61 mm^−1^
*T* = 113 (2) K
0.12 × 0.10 × 0.08 mm

#### Data collection

Rigaku Saturn diffractometer
Absorption correction: multi-scan (*CrystalClear*; Rigaku/MSC, 2005 ▶) *T*_min_ = 0.930, *T*_max_ = 0.953
5459 measured reflections
2858 independent reflections
2636 reflections with *I* > 2σ(*I*)
*R*_int_ = 0.028

#### Refinement

*R*[*F* ^2^ > 2σ(*F* ^2^)] = 0.024
*wR*(*F* ^2^) = 0.060
*S* = 1.06
2858 reflections
179 parameters
1 restraint
H atoms treated by a mixture of independent and constrained refinement
Δρ_max_ = 0.21 e Å^−3^
Δρ_min_ = −0.23 e Å^−3^
Absolute structure: Flack (1983 ▶), 996 Friedel pairs
Flack parameter: 0.04 (4)

### 

Data collection: *CrystalClear* (Rigaku/MSC, 2005 ▶); cell refinement: *CrystalClear*; data reduction: *CrystalClear*; program(s) used to solve structure: *SHELXS97* (Sheldrick, 2008 ▶); program(s) used to refine structure: *SHELXL97* (Sheldrick, 2008 ▶); molecular graphics: *SHELXTL* (Sheldrick, 2008 ▶); software used to prepare material for publication: *SHELXTL*.

## Supplementary Material

Crystal structure: contains datablocks I, global. DOI: 10.1107/S1600536808005369/hg2380sup1.cif

Structure factors: contains datablocks I. DOI: 10.1107/S1600536808005369/hg2380Isup2.hkl

Additional supplementary materials:  crystallographic information; 3D view; checkCIF report

## Figures and Tables

**Table 1:** Hydrogen-bond geometry (Å, °)

*D*—H⋯*A*	*D*—H	H⋯*A*	*D*⋯*A*	*D*—H⋯*A*
O1—H1⋯O3^i^	0.89 (3)	1.99 (3)	2.8095 (18)	152 (3)
O1—H1⋯Cl1^i^	0.89 (3)	2.92 (3)	3.6201 (16)	136 (2)
N1—H1*A*⋯O3^ii^	0.95 (3)	1.87 (3)	2.8085 (19)	170 (2)
N1—H1*B*⋯O2^iii^	0.94 (2)	1.87 (2)	2.796 (2)	165.1 (19)
N1—H1*B*⋯Cl2^iii^	0.94 (2)	2.94 (2)	3.5647 (16)	124.5 (16)

Symmetry codes: (i) 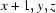; (ii) 
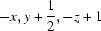
; (iii) 
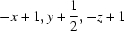
.

**Fig. 1.**
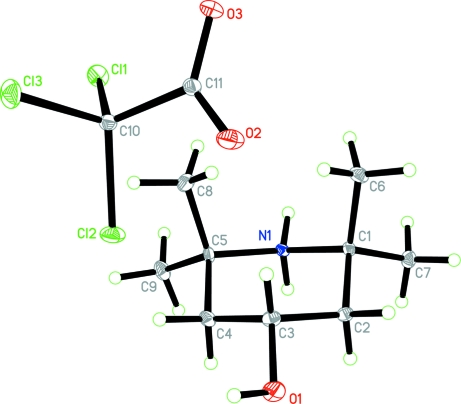
Crystal structure of the title compound with atom labeling and displacement ellipsoids drawn at the 30% probability level. H atoms are shown as small spheres of arbitrary radii.

## supplementary crystallographic information

### Comment

The title compound was obtained as a byproduct in the synthesis of hindered amine light stabilizers preventing the degradation of polyolefins in sunlight, in which 2,2,6,6-tetramethylpiperidin-4-ol is a very important intermediate (Borzatta & Carrozza,1991). We report here the crystal structure 4-hydroxy-2,2,6,6-tetramethylpiperidinium trichloroacetate (Fig. 1). Intermolecular O—H···O, N—H···O, O—H···Cl, N—H···Cl hydrogen bonds are observed which help to establish the crystal packing. The piperidine ring adopts a chair conformation.

### Experimental

0.25 g (1.6 mmol) of 2,2,6,6-tetramethylpiperidin-4-ol was dissolved in 3.2 ml of trichloroacetate acid solution (1.6 mmol, 0.26 g). Colorless crystals of the title compound were obtained by slow evaporation of the solvent.

### Refinement

All H atoms bound to C atoms were constrained; positioned geometrically (C—H = 0.96–0.98 Å) and refined as riding with *U*_iso_(H)=1.2*U*_eq_(carrier) or 1.5_eq_(methyl groups). H atoms of O—H and N—H were located from difference maps and then refined freely.

### Crystal data

**Table:**

C_9_H_20_NO^+^·C_2_Cl_3_O_2_^–^	*F*_000_ = 336
*M**_r_* = 320.63	*D*_x_ = 1.419 Mg m^−^^3^
Monoclinic, *P*2_1_	Mo *K*α radiation λ = 0.71073 Å
Hall symbol: P 2yb	Cell parameters from 2559 reflections
*a* = 6.3468 (13) Å	θ = 1.4–27.9º
*b* = 14.450 (3) Å	µ = 0.61 mm^−^^1^
*c* = 8.2175 (16) Å	*T* = 113 (2) K
β = 95.19 (3)º	Block, colorless
*V* = 750.5 (3) Å^3^	0.12 × 0.10 × 0.08 mm
*Z* = 2	

### Data collection

**Table:**

Rigaku Saturn diffractometer	2858 independent reflections
Radiation source: rotating anode	2636 reflections with *I* > 2σ(*I*)
Monochromator: confocal	*R*_int_ = 0.028
*T* = 113(2) K	θ_max_ = 27.9º
ω and φ scans	θ_min_ = 2.5º
Absorption correction: multi-scan(CrystalClear; Rigaku/MSC, 2005)	*h* = −8→8
*T*_min_ = 0.930, *T*_max_ = 0.953	*k* = −15→19
5459 measured reflections	*l* = −10→10

### Refinement

**Table:**

Refinement on *F*^2^	Hydrogen site location: inferred from neighbouring sites
Least-squares matrix: full	H atoms treated by a mixture of independent and constrained refinement
*R*[*F*^2^ > 2σ(*F*^2^)] = 0.024	*w* = 1/[σ^2^(*F*_o_^2^) + (0.0324*P*)^2^] where *P* = (*F*_o_^2^ + 2*F*_c_^2^)/3
*wR*(*F*^2^) = 0.060	(Δ/σ)_max_ = 0.001
*S* = 1.06	Δρ_max_ = 0.21 e Å^−^^3^
2858 reflections	Δρ_min_ = −0.23 e Å^−^^3^
179 parameters	Extinction correction: none
1 restraint	Absolute structure: Flack (1983), 996 Friedel pairs
Primary atom site location: structure-invariant direct methods	Flack parameter: 0.04 (4)
Secondary atom site location: difference Fourier map	

### Special details

**Table:**

Geometry. All e.s.d.'s (except the e.s.d. in the dihedral angle between two l.s. planes) are estimated using the full covariance matrix. The cell e.s.d.'s are taken into account individually in the estimation of e.s.d.'s in distances, angles and torsion angles; correlations between e.s.d.'s in cell parameters are only used when they are defined by crystal symmetry. An approximate (isotropic) treatment of cell e.s.d.'s is used for estimating e.s.d.'s involving l.s. planes.
Refinement. Refinement of *F*^2^ against ALL reflections. The weighted *R*-factor *wR* and goodness of fit *S* are based on *F*^2^, conventional *R*-factors *R* are based on *F*, with *F* set to zero for negative *F*^2^. The threshold expression of *F*^2^ > σ(*F*^2^) is used only for calculating *R*-factors(gt) *etc*. and is not relevant to the choice of reflections for refinement. *R*-factors based on *F*^2^ are statistically about twice as large as those based on *F*, and *R*- factors based on ALL data will be even larger.

### Fractional atomic coordinates and isotropic or equivalent isotropic displacement parameters (Å^2^)

**Table:**

	*x*	*y*	*z*	*U*_iso_*/*U*_eq_	
Cl1	−0.04464 (7)	0.51471 (3)	0.10942 (5)	0.01980 (10)	
Cl2	0.34863 (6)	0.41431 (3)	0.12885 (6)	0.02203 (11)	
Cl3	−0.00077 (8)	0.35637 (3)	−0.09864 (5)	0.02572 (11)	
O1	0.7973 (2)	0.47566 (9)	0.51619 (17)	0.0227 (3)	
H1	0.798 (4)	0.453 (2)	0.415 (4)	0.063 (10)*	
O2	0.1032 (2)	0.27147 (9)	0.27469 (18)	0.0247 (3)	
O3	−0.20242 (18)	0.34925 (9)	0.25644 (15)	0.0169 (3)	
N1	0.4803 (2)	0.71305 (9)	0.64562 (18)	0.0111 (3)	
C1	0.4608 (3)	0.62855 (12)	0.75507 (19)	0.0129 (3)	
C2	0.6286 (3)	0.55858 (11)	0.7135 (2)	0.0139 (3)	
H2A	0.7701	0.5832	0.7515	0.017*	
H2B	0.6080	0.5005	0.7739	0.017*	
C3	0.6235 (3)	0.53661 (11)	0.5324 (2)	0.0159 (4)	
H3	0.4876	0.5051	0.4945	0.019*	
C4	0.6475 (3)	0.62493 (13)	0.4346 (2)	0.0164 (3)	
H4A	0.6371	0.6090	0.3170	0.020*	
H4B	0.7905	0.6506	0.4638	0.020*	
C5	0.4836 (3)	0.69993 (12)	0.46158 (19)	0.0135 (3)	
C6	0.2366 (3)	0.58770 (13)	0.7353 (2)	0.0198 (4)	
H6A	0.1327	0.6377	0.7379	0.030*	
H6B	0.2191	0.5443	0.8248	0.030*	
H6C	0.2149	0.5550	0.6306	0.030*	
C7	0.5076 (3)	0.66393 (13)	0.9293 (2)	0.0189 (4)	
H7A	0.6495	0.6912	0.9419	0.028*	
H7B	0.5003	0.6124	1.0061	0.028*	
H7C	0.4029	0.7110	0.9519	0.028*	
C8	0.2629 (3)	0.67658 (13)	0.3825 (2)	0.0198 (4)	
H8A	0.1595	0.7193	0.4228	0.030*	
H8B	0.2266	0.6130	0.4104	0.030*	
H8C	0.2609	0.6825	0.2636	0.030*	
C9	0.5521 (3)	0.79290 (13)	0.3950 (2)	0.0213 (4)	
H9A	0.4450	0.8399	0.4123	0.032*	
H9B	0.5676	0.7871	0.2779	0.032*	
H9C	0.6878	0.8113	0.4523	0.032*	
C10	0.0700 (3)	0.40324 (12)	0.09850 (19)	0.0130 (3)	
C11	−0.0172 (3)	0.33454 (11)	0.2249 (2)	0.0133 (3)	
H1A	0.373 (4)	0.7546 (18)	0.674 (3)	0.037 (7)*	
H1B	0.610 (4)	0.7410 (15)	0.683 (3)	0.025 (6)*	

### Atomic displacement parameters (Å^2^)

**Table:**

	*U*^11^	*U*^22^	*U*^33^	*U*^12^	*U*^13^	*U*^23^
Cl1	0.0227 (2)	0.01408 (19)	0.0232 (2)	0.00440 (16)	0.00505 (17)	0.00388 (16)
Cl2	0.01100 (19)	0.0235 (2)	0.0318 (3)	−0.00221 (15)	0.00291 (17)	0.00920 (19)
Cl3	0.0321 (3)	0.0304 (3)	0.0149 (2)	−0.0025 (2)	0.00289 (18)	−0.00596 (17)
O1	0.0249 (7)	0.0194 (7)	0.0241 (7)	0.0108 (5)	0.0033 (6)	−0.0052 (6)
O2	0.0147 (6)	0.0220 (7)	0.0377 (8)	0.0023 (5)	0.0044 (6)	0.0151 (6)
O3	0.0125 (6)	0.0160 (6)	0.0230 (7)	−0.0007 (5)	0.0056 (5)	−0.0007 (5)
N1	0.0114 (7)	0.0098 (7)	0.0124 (7)	0.0007 (5)	0.0033 (6)	−0.0002 (5)
C1	0.0127 (8)	0.0130 (8)	0.0133 (8)	0.0010 (6)	0.0032 (6)	0.0029 (6)
C2	0.0137 (8)	0.0124 (8)	0.0156 (9)	0.0027 (6)	0.0014 (7)	0.0001 (6)
C3	0.0152 (8)	0.0131 (8)	0.0193 (9)	0.0035 (6)	0.0010 (7)	−0.0028 (6)
C4	0.0164 (9)	0.0187 (8)	0.0149 (8)	0.0020 (6)	0.0049 (7)	−0.0023 (7)
C5	0.0167 (8)	0.0153 (8)	0.0089 (7)	0.0010 (6)	0.0031 (6)	0.0010 (6)
C6	0.0152 (9)	0.0174 (9)	0.0276 (10)	−0.0007 (7)	0.0059 (8)	0.0059 (7)
C7	0.0228 (9)	0.0215 (10)	0.0127 (8)	0.0047 (7)	0.0028 (7)	0.0000 (7)
C8	0.0196 (9)	0.0215 (9)	0.0176 (9)	−0.0002 (7)	−0.0023 (7)	0.0013 (7)
C9	0.0272 (11)	0.0164 (9)	0.0214 (10)	−0.0001 (7)	0.0090 (8)	0.0043 (7)
C10	0.0118 (8)	0.0134 (8)	0.0141 (8)	0.0009 (6)	0.0021 (6)	0.0010 (6)
C11	0.0124 (8)	0.0145 (8)	0.0130 (8)	−0.0019 (6)	0.0010 (6)	−0.0006 (6)

### Geometric parameters (Å, °)

**Table:**

Cl1—C10	1.7729 (17)	C4—C5	1.532 (2)
Cl2—C10	1.7710 (17)	C4—H4A	0.9900
Cl3—C10	1.7756 (17)	C4—H4B	0.9900
O1—C3	1.427 (2)	C5—C8	1.528 (2)
O1—H1	0.89 (3)	C5—C9	1.529 (3)
O2—C11	1.235 (2)	C6—H6A	0.9800
O3—C11	1.245 (2)	C6—H6B	0.9800
N1—C5	1.526 (2)	C6—H6C	0.9800
N1—C1	1.528 (2)	C7—H7A	0.9800
N1—H1A	0.95 (3)	C7—H7B	0.9800
N1—H1B	0.94 (2)	C7—H7C	0.9800
C1—C7	1.524 (2)	C8—H8A	0.9800
C1—C2	1.529 (2)	C8—H8B	0.9800
C1—C6	1.535 (2)	C8—H8C	0.9800
C2—C3	1.519 (2)	C9—H9A	0.9800
C2—H2A	0.9900	C9—H9B	0.9800
C2—H2B	0.9900	C9—H9C	0.9800
C3—C4	1.523 (2)	C10—C11	1.573 (2)
C3—H3	1.0000		
			
C3—O1—H1	112.3 (19)	C8—C5—C4	113.00 (15)
C5—N1—C1	119.53 (13)	C9—C5—C4	110.55 (15)
C5—N1—H1A	113.1 (15)	C1—C6—H6A	109.5
C1—N1—H1A	105.4 (15)	C1—C6—H6B	109.5
C5—N1—H1B	106.5 (14)	H6A—C6—H6B	109.5
C1—N1—H1B	105.4 (13)	C1—C6—H6C	109.5
H1A—N1—H1B	106 (2)	H6A—C6—H6C	109.5
C7—C1—N1	105.40 (13)	H6B—C6—H6C	109.5
C7—C1—C2	110.56 (14)	C1—C7—H7A	109.5
N1—C1—C2	107.56 (13)	C1—C7—H7B	109.5
C7—C1—C6	109.16 (14)	H7A—C7—H7B	109.5
N1—C1—C6	111.62 (14)	C1—C7—H7C	109.5
C2—C1—C6	112.31 (14)	H7A—C7—H7C	109.5
C3—C2—C1	113.80 (14)	H7B—C7—H7C	109.5
C3—C2—H2A	108.8	C5—C8—H8A	109.5
C1—C2—H2A	108.8	C5—C8—H8B	109.5
C3—C2—H2B	108.8	H8A—C8—H8B	109.5
C1—C2—H2B	108.8	C5—C8—H8C	109.5
H2A—C2—H2B	107.7	H8A—C8—H8C	109.5
O1—C3—C2	105.80 (14)	H8B—C8—H8C	109.5
O1—C3—C4	110.67 (14)	C5—C9—H9A	109.5
C2—C3—C4	110.33 (14)	C5—C9—H9B	109.5
O1—C3—H3	110.0	H9A—C9—H9B	109.5
C2—C3—H3	110.0	C5—C9—H9C	109.5
C4—C3—H3	110.0	H9A—C9—H9C	109.5
C3—C4—C5	114.51 (14)	H9B—C9—H9C	109.5
C3—C4—H4A	108.6	C11—C10—Cl2	111.74 (11)
C5—C4—H4A	108.6	C11—C10—Cl1	111.69 (11)
C3—C4—H4B	108.6	Cl2—C10—Cl1	108.65 (9)
C5—C4—H4B	108.6	C11—C10—Cl3	106.67 (11)
H4A—C4—H4B	107.6	Cl2—C10—Cl3	109.25 (9)
N1—C5—C8	110.77 (14)	Cl1—C10—Cl3	108.77 (9)
N1—C5—C9	105.97 (14)	O2—C11—O3	128.65 (16)
C8—C5—C9	108.77 (14)	O2—C11—C10	116.15 (14)
N1—C5—C4	107.55 (13)	O3—C11—C10	115.14 (14)
			
C5—N1—C1—C7	168.77 (14)	C1—N1—C5—C9	−167.95 (14)
C5—N1—C1—C2	50.78 (19)	C1—N1—C5—C4	−49.69 (19)
C5—N1—C1—C6	−72.85 (19)	C3—C4—C5—N1	50.25 (19)
C7—C1—C2—C3	−166.72 (14)	C3—C4—C5—C8	−72.32 (19)
N1—C1—C2—C3	−52.12 (18)	C3—C4—C5—C9	165.51 (16)
C6—C1—C2—C3	71.08 (18)	Cl2—C10—C11—O2	−29.86 (19)
C1—C2—C3—O1	176.40 (14)	Cl1—C10—C11—O2	−151.81 (14)
C1—C2—C3—C4	56.67 (19)	Cl3—C10—C11—O2	89.47 (17)
O1—C3—C4—C5	−172.59 (14)	Cl2—C10—C11—O3	152.80 (13)
C2—C3—C4—C5	−55.84 (19)	Cl1—C10—C11—O3	30.85 (18)
C1—N1—C5—C8	74.25 (18)	Cl3—C10—C11—O3	−87.87 (15)

### Hydrogen-bond geometry (Å, °)

**Table:**

*D*—H···*A*	*D*—H	H···*A*	*D*···*A*	*D*—H···*A*
O1—H1···O3^i^	0.89 (3)	1.99 (3)	2.8095 (18)	152 (3)
O1—H1···Cl1^i^	0.89 (3)	2.92 (3)	3.6201 (16)	136 (2)
N1—H1A···O3^ii^	0.95 (3)	1.87 (3)	2.8085 (19)	170 (2)
N1—H1B···O2^iii^	0.94 (2)	1.87 (2)	2.796 (2)	165.1 (19)
N1—H1B···Cl2^iii^	0.94 (2)	2.94 (2)	3.5647 (16)	124.5 (16)

Symmetry codes: (i) *x*+1, *y*, *z*; (ii) −*x*, *y*+1/2, −*z*+1; (iii) −*x*+1, *y*+1/2, −*z*+1.
